# Mapping Microplastic Movement: A Phase Diagram to Predict Nonbuoyant Microplastic Modes of Transport at the Particle Scale

**DOI:** 10.1021/acs.est.4c08128

**Published:** 2024-09-28

**Authors:** Hadeel Al-Zawaidah, Merel Kooi, Ton Hoitink, Bart Vermeulen, Kryss Waldschläger

**Affiliations:** † Hydrology and Environmental Hydraulics Group, Wageningen University and Research, 6700 AA Wageningen, The Netherlands; ‡ Aquatic Ecology and Water Quality Management Group, Wageningen University and Research, 6700 AA Wageningen, The Netherlands

**Keywords:** microplastic transport, transport stage, phase diagram, bedload transport, suspension

## Abstract

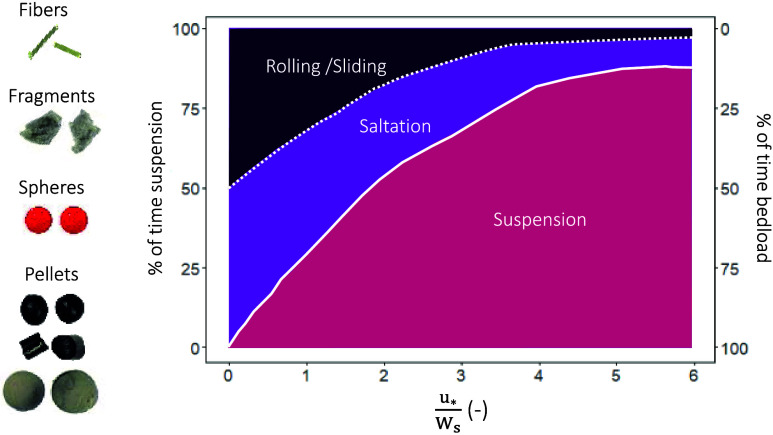

Microplastics pose numerous threats to aquatic environments, yet understanding their transport mechanisms remains limited. Drawing from natural sediment research provides valuable insights to address this knowledge gap. One key dimensionless number used to describe sediment transport is the transport stage, referring to the ratio between the flow shear velocity and the particle settling velocity. However, variations in physical properties, such as shape and density, raise concerns about the applicability of existing sediment transport theories to microplastics. To address this challenge, we employed a physical modeling approach, examining 24 different nonbuoyant microplastic particles in a turbulent open channel flow. Utilizing 3D particle tracking, a total of 720 trajectories were recorded and analyzed. Microplastic particles exhibited transport modes akin to natural sediments, including rolling/sliding, saltation, and suspension. The transport stage strongly correlated with these modes, as well as with the mean forward velocity and mean position in the water column. Notably, particle shape emerged as a critical factor influencing transport dynamics. Due to their lower settling velocity, fibers tended to stay closer to the water surface with lower forward velocities compared to spheres. Based on the laboratory results, a new phase diagram for microplastics is introduced analogous to an existing diagram for sediments.

## Abstract 2

This study uncovers similarities between nonbuoyant microplastic and sediment transport, highlighting the significance of particle shape in microplastic transport dynamics. The findings led to a novel nonbuoyant plastic transport phase diagram.

## 1 Introduction

Microplastics are abundant in aquatic environments, posing a pervasive environmental concern.^1−3^ In response, various initiatives, spanning legislative, scientific, and social realms, target reduced microplastic emissions and the removal of environmental microplastics.^4,5^ However, our ability to effectively assess and monitor the outcomes of these efforts is impeded by multiple knowledge gaps.^6^ Rivers are a critical area of focus as they can act as both potential sinks^7^ and important routes for microplastics to the ocean.^8−11^ Despite their importance, the mechanisms governing riverine microplastic transport and abundance remain poorly understood.^12^ Closing these knowledge gaps is needed to evaluate and optimize mitigation strategies and enhance risk assessments.

Flow characteristics can influence the transport of microplastic particles in rivers in a manner comparable to other natural riverine components (e.g., sediment, organic matter and air bubbles).^13−15^ Recently, there has been growing interest in studying how the fundamental principles of flow-sediment interactions apply to microplastics. The Rouse model has been investigated to describe the vertical distribution of microplastics.^13,16−18^ However, different densities and particle shapes challenge the direct adoption of sediment transport equations and models. For instance, the settling velocity and the critical shear velocity dictating the incipient motion of microplastics differed from theoretical values based on sediment transport equations.^15,19,20^ Lofty et al.^14^ investigated the modes of transport experienced by perfect spheres with size and density ranges comparable to nonbuoyant microplastics (i.e., suspension or bedload transport). They showed that the probability of saltation and rolling was governed by the Rouse number. However, recent investigation with fragmented macroplastics revealed a deviation from the predicted Rouse profile due to the particle-bed dynamics.^21^ These findings highlight the need for further research into the effects of particle shape on microplastic modes of transport.

Here, we further investigate the modes of transport of nonbuoyant microplastic particles with different shapes, including fibers, fragments, pellets and spheres, under different turbulent flow conditions. Guided by the well-established practice in natural sediment,^22−24^ we focus on the transport stage, describing the balance between turbulence shear forces pushing particles upward in the water column (represented by flow shear velocity, u_*_) and gravitational forces driving particles downward (represented by particle settling velocity, *W*_s_). We investigate the transport stage as a mean to better describe and predict abundance locations of microplastic particles in the water column, as well as the transport rate and the modes of transport which can describe their movement. It should be noted that we choose the transport stage over the Rouse number due to our focus on near-bed dynamics rather than the concentration-driven mixing described by the Rouse model.

Our primary objective is to determine how well the transport stage describes microplastic movement. We analyze trajectory characteristics, specifically the mean position in the water column (*Z*_P_) and the transport velocity of plastic particles with the flow (*U*_P_), to identify different transport modes for nonbuoyant microplastics. Based on our laboratory experiments, we developed a new phase diagram to predict these transport modes depending on the transport stage. This work provides crucial insights for developing effective mitigation measures and predicting transport rates of nonbuoyant microplastics in river environments.

## 2 Material and Methods

### 2.1 Material Matrix

**Figure 1:**
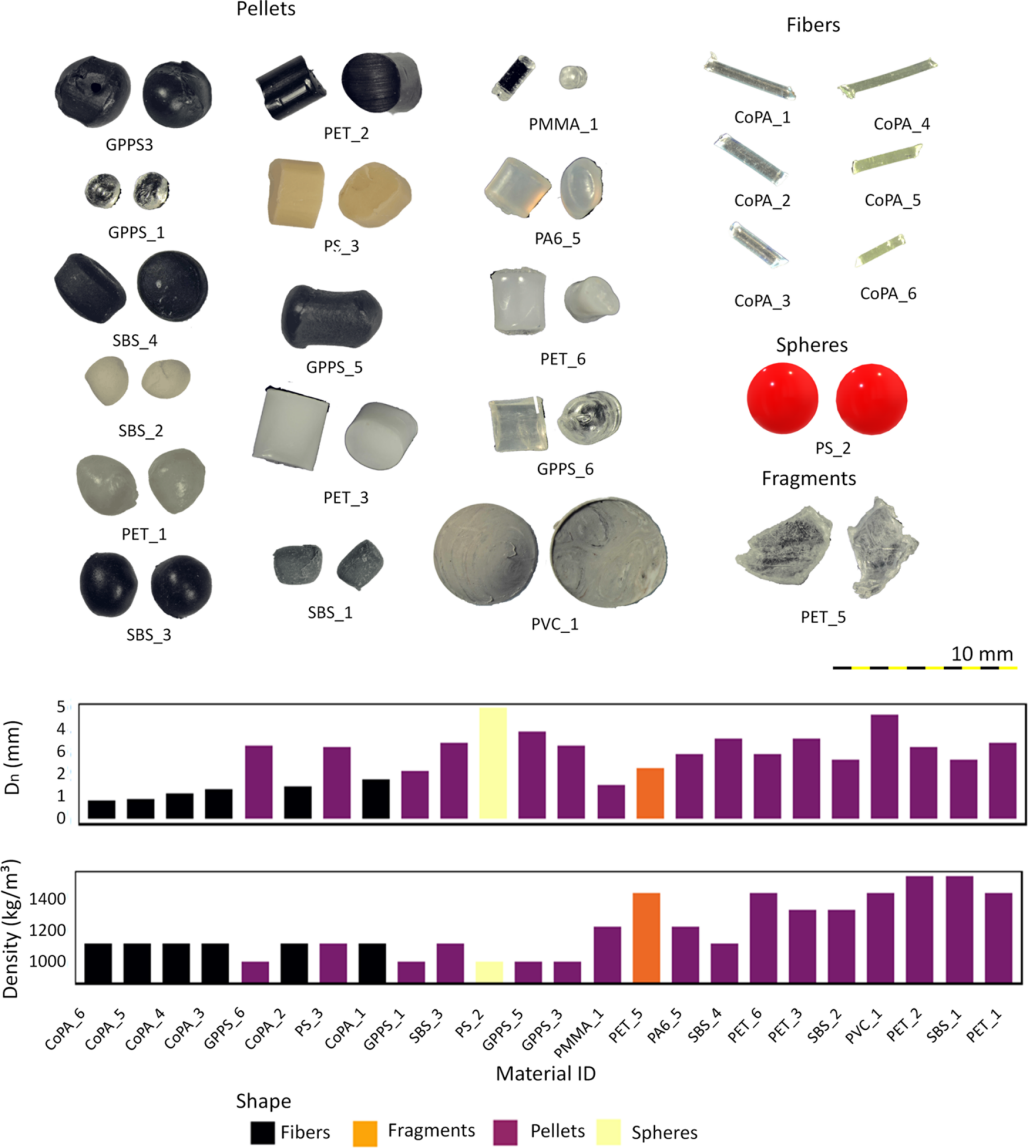
An overview of the tested microplastic particles properties (further information can be found in Supporting Information).

To capture the diversity in microplastic characteristics, while still maintaining a feasible experimental program, 24 different microplastic particles were examined (Figure 1). The selection of microplastics was based on the types of polymers and shapes predominantly found in riverine systems.^25−27^ The particles covered a density range between 1.09 and 1.49 g cm^–3^. Particle sizes ranged from 0.5 to 5 mm, which is the commonly used upper size limit for microplastics.^28^ Despite the presence of smaller microplastics in the environment, 0.5 mm was the smallest size detectable by the used cameras. The selected microplastics included a variety of shapes including spheres, fragments, pellets (i.e., cylinders and ellipsoids), and fibers. It should be noted here that the fibers used in the present study are thicker (diameter: 0.5–1 mm) than usual clothing fibers. The particles were coated with a thin layer of water-based ink to improve their visibility. Using a coating is becoming more common for detection and transport studies.^29^ The used coating did not significantly change the particle density. However, it minimized variations in affinity to air bubbles and water among different polymers, which may cause a deviated actual particle settling velocity from the theoretically calculated value.^30^ Hence, the coating facilitated isolating the effects of particle shape, size, and density.

In preliminary experiments, additional buoyant plastics with a density range between 0.89 and 1 g cm^–3^, and shape and size ranges comparable to the tested nonbuoyant microplastics were investigated. Under the studied flow velocities, the buoyant microplastics remained near the surface regardless of their characteristics. This behavior occurred due to the different dynamics governing buoyant versus nonbuoyant microplastic transport. Nonbuoyant microplastics tend to settle toward the bed and are influenced by the bed boundary layer and associated bed shear forces, while buoyant microplastics float near the surface and are affected by surface-driven phenomena like surface tension and wind.^31,32^ Therefore, we decided to focus on nonbuoyant particles in this study.

To capture the diversity and complexity of the particle shapes, dimensionless shape descriptors proposed by Van Melkebeke et al.^20^ were used (Table 1). Particle classification involved two steps. First, the three principal dimensions (L, I, S) were determined for each particle using ImageJ (Table 1). A caliper was used to determine the third dimension whenever the particle geometry did not allow for adjusting the orientation of the particles in the images to determine the third dimension. In both cases, these three dimensions were determined based on the average of 10 measurements per microplastic type, following the work of Kerpen et al.^33^ The percentage standard deviation was reported for the three principal dimensions (, where *X̅* is the mean of the measurements of the dimension of interest). Then, the particle volume (*V*_p_) and density were determined using a UltraPyc 1200e Powder density meter. The three principal dimensions and *V*_p_ were used to calculate the aspect ratio (φ), Corey shape factor (CSF), flatness (F), sphericity (ϕ) and elongation (*e*). Further, the settling velocities of the particles were calculated following the equations developed by Waldschläger & Schüttrumpf.^15^ This method in determining the settling velocity was based on information availability regarding the particle shape and its proven reliability compared to other methods^20^ for the range of particles used in the present study. Therefore, the nominal particle diameter was determined (*d*_*n*_) using the equation in Table 1, which was the input parameter for the settling velocity calculations. Additionally, the particle density and size were characterized using the dimensionless particle diameter, D_*_, defined as
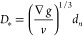
1where ν is the kinematic viscosity of the fluid (m^2^ s^–1^) and ∇ is the ratio of excess particle density to water density (−), defined as
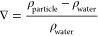
2

**Table 1:** Summary of the Descriptive Shape Factors Adopted for the Present Study

shape factors						
Corey shape factor (CSF)	sphericity (ϕ)	elongation (*e*)	flatness (F)	aspect ratio (φ)		nominal diameter (*d*_*n*_)
					

input parameters		
description	symbol	formula/measurement method
orthogonal longest axis (mm)	*L*	measured using ImageJ
intermediate axis (mm)	*I*	measured using ImageJ
shortest axis (mm)	*S*	measured using a caliper or ImageJ
the measured volume of the particle (mm^3^)	*V*_p_	measured with UltraPyc 1200e powder density meter.
the diameter of an equivalent sphere (mm)	*d*_p_	
the surface area of the equivalent sphere (mm^2^)	*A*_sph_	
the surface area of the particle (mm^2^)	*A*_p_	calculated as 2π*rh* + 2π*r*^2^ for perfect cylinders and as for the remaining particles

This parameter is used both in sediment and in microplastic analyses.^15,34−36^ Using D_*_ in the present study was necessary to understand the combined effect of size and density as it was not possible to isolate them due to the experimental matrix complexities.

### 2.2 Experimental Setup

**Figure 2:**
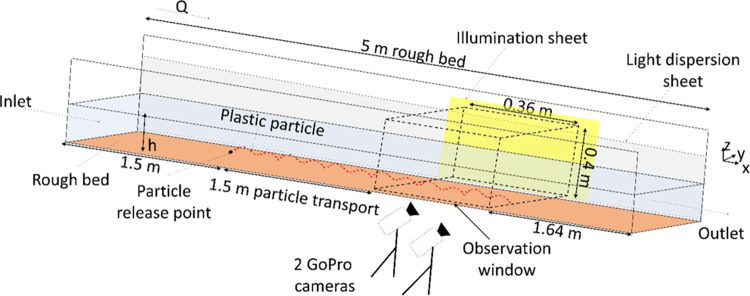
Layout of the experimental setup (distances not to scale).

A 5 m long recirculating flume with a cross-section of 450 mm by 300 mm at the Kraijenhoff van de Leur Laboratory at Wageningen University was used for the experiments. The flume, with a maximum discharge of 40 l s^–1^ and an adjustable slope (Figure 2), allows water depth control via a downstream vertical gate. The experiments employed a fixed flume bed, with a layer of fine sand (1.85 mm diameter) glued to the bed to introduce roughness for fully developed turbulence. Microplastic transport was assessed under three subcritical turbulent flow conditions (Table 2), representative of flows comsmonly occurring in natural rivers. The flow velocity profiles in two dimensions (parallel to the flow (*u*), and orthogonal to the flume bed (*w*)) were obtained at the centerline of the effective section using a UB-Lab 2C velocity vector profiler (ADVP). Instantaneous velocity measurements were used to obtain the bed shear velocity (u_*_).

**Table 2:** Overview of Flow Conditions within the Experimental Setup^a^

flow condition	discharge	water depth	mean velocity	Reynolds number	Froude number	shear velocity	bed slope
**ID**	*Q* (l s^–1^)	*h* (cm)	*U* (m s^–1^)	Re (−)	Fr (−)	u_*_ (m s^–1^)	*s* (−)
F1	40.0	17.4	0.83	14,442	0.635	0.103	0.15%
F2	25.0	23.0	0.47	10,810	0.313	0.051	0.15%
F3	15.0	24.3	0.31	7533	0.201	0.018	0.15%

a Where: *u*_*i*_ is the velocity at point *i* relative to the water depth (*Z*), *u* is the kinematic viscosity of water (m^2^ s^–1^), *g* is gravitational acceleration (m s^–2^), ρ is water density (kg m^–3^), τ_w_ is the wall shear stress (N m^–2^)

### 2.3 Loading Regime

Microplastics were introduced 1.5 m from the flume inlet at the centerline of the flume bed. Given the density of the microplastics (>1 g cm^–3^), they were released near the flume bed, reflecting their terminal position relative to settling velocities, using a 3 cm wide, 30 cm long stainless-steel clamp to minimize flow interference. The particles were transported freely for 1.5 m before reaching the observation window, where their transport trajectory was recorded. This approach ensured that the particles attained equilibrium before tracking their transport and minimized the influences of the flume inlet and outlet. Prior to injection into the flume, the particles underwent water soaking to prevent air bubbles from attaching to their surfaces.

### 2.4 Particle Tracking Setup and Analysis

The transport of the microplastic particles was recorded using a particle tracking photogrammetry setup (PTV). The setup included two GoPro Hero 11 cameras, which allowed for collecting videos with a frame rate of 50 fps and 5.3 K resolution. The cameras faced one side of the flume wall, covering a 0.36 m observation window parallel (5312 pixels) and 0.3 m perpendicular (2933 pixels) to the flow direction. To improve the contrast between the tracked particles and the background, the roughened flume bed was sprayed with a thin layer of white paint. Further, the observation window was illuminated using a light source facing the cameras, which was evenly distributed through a dispersive white sheet. The use of two synchronized cameras allowed for generating the 3D trajectory of the particles, by employing the concepts of epipolar distance and stereoscopy.^37^ Although both cameras started recording simultaneously using a remote control, further postprocessing was needed to ensure that frames obtained from both cameras were in synchronization. This was achieved through postprocessing based on a distinctive sound at the beginning of the video recording. Camera calibration was performed using a 3D calibration grid in the observation window at a known distance relative to the flume coordinate system.

The trajectories of the particles were obtained using stereoscopic reconstruction following Douxchamps et al.^38^ Videos were processed with an in-house MATLAB code to convert frames from RGB to grayscale, to perform foreground detection, and to determine particle centroids relative to the camera’s 2D coordinate system in each frame. Noise elimination was achieved by cropping the frame to the area of interest. Then, the 2D camera coordinates were translated to real-world 3D coordinates following Spinewine et al.^39^ Although refraction was not accounted for, low camera angles were maintained to minimize its effects.^40^ Validated by reconstructing reference points within the observation window, the system achieved ±5 mm accuracy, which is sufficient for the present study objectives.

A total of 720 individual trajectories were obtained, including 24 microplastic particles under 3 different flow conditions, with 10 repetitions per particle aligning with established practices in literature.^14,19^ Each trajectory included instantaneous measurements of velocity and position, each representing a statistically independent event. The study focused on three transport modes: rolling/sliding, saltation, and suspension, mirroring key modes identified in sedimentology.^22,24^ In classical sediment studies, the analysis of particle transport trajectories traditionally focuses on suspended sediments.^24,41^ We adopted a similar approach to define the particle’s mean forward velocity and position. The mean forward velocity (*U*_p_) is the average particle velocity component parallel to the primary flow direction (*u*), calculated as
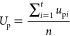
3where *u*_pi_ is the recorded instantaneous velocity at time step *i* and *n* is the number of measurements. Similarly, the mean position in the water column relative to the bed (*Z*_p_) is the average particle position within the water column relative to the flume bed, measured as
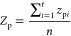
4where *z*_p*i*_ is the recorded instantaneous particle positions at time step *i* and *n* is the number of measurements. This approach allows for predicting the overall microplastic transport rate and occurrence relative to the water column regardless of their modes of transport. It should be noted here that the analyzed trajectory for each particle can be divided into intervals of different modes of transport (i.e., saltation, rolling, and sliding), as explained in the following paragraph, where the mean forward velocity may vary for each of these intervals. However, these variations are limited to ±25%, as demonstrated by Abbott and Francis,^22^ and are therefore limited relative to the mean forward velocity across the full trajectory. Test reproducibility was evaluated by determining the relative error for mean particle position and forward velocity for each flow-particle combination.

**Figure 3:**
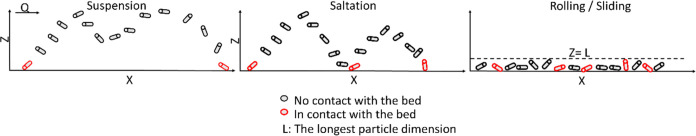
Conceptual diagram illustrating the criteria for determining the particle modes of transport.

The individual modes were determined based on analyzing the full particle trajectory following predefined criteria (Figure 3). If all measurement points between two points of contact with the bed were below the longest particle dimension (*L*), the particle was classified as rolling/sliding. Trajectories without local minima but with one or more local maxima were categorized as saltation, i.e., the transport mode where the particle bounces consistently over the bed similar to a stone bouncing over the water surface. In cases where both local maxima and minima occurred consecutively within the water column, the particles were considered to be in suspension. What differentiates suspension from saltation is that particles in suspension exhibit both peaks and troughs in their trajectories within the water column between two moments of contact with the bed. Then, the full trajectory was segmented into intervals corresponding to each transport mode. These segments were translated into a percentage of time relative to the total duration it took for the particle to pass through the observation window. A MATLAB code was developed to streamline the trajectory analysis. While this approach effectively addressed spherical and semispherical particles, additional visual inspection was necessary to filter out noise observations due to particle shapes. This noise included multiple local maxima and minima in trajectories caused by particle rotation and changes in the dimension facing the cameras. Moreover, this step proved crucial for rectifying errors in estimating particle locations due to inaccuracies caused by the physical limitations of the setup.

Following the video analysis process, the impact of particle characteristics on the trajectories was examined through principal component analysis (PCA) and a multivariable ANOVA test. The PCA was utilized to determine the overall structure and patterns in the data, and to determine the variables describing most of the variances. The multivariable ANOVA test provided information about the statistical significance of each variable. Together, these tests provided comprehensive understanding of the underlying structure and the key variables governing the variations in the data set. The tests were performed using R studio and the detailed results can be found in the Supporting Information. The particle shape was evaluated using the five descriptive shape factors introduced earlier, while D_*_ was used to examine the combined effect of size and density.

## 3 Results and Discussion

The findings of the present study are outlined in two subsections. In the particle trajectory section, results regarding the mean forward velocity and mean particle position are presented, focusing on the test reproducibility, the impact of the transport stage and the effect of the particle characteristics. The next section addresses the impact of particle characteristics and transport stage on the modes of transport.

### 3.1 Particle Trajectory

#### 3.1.1 Test Reproducibility

**Figure 4:**
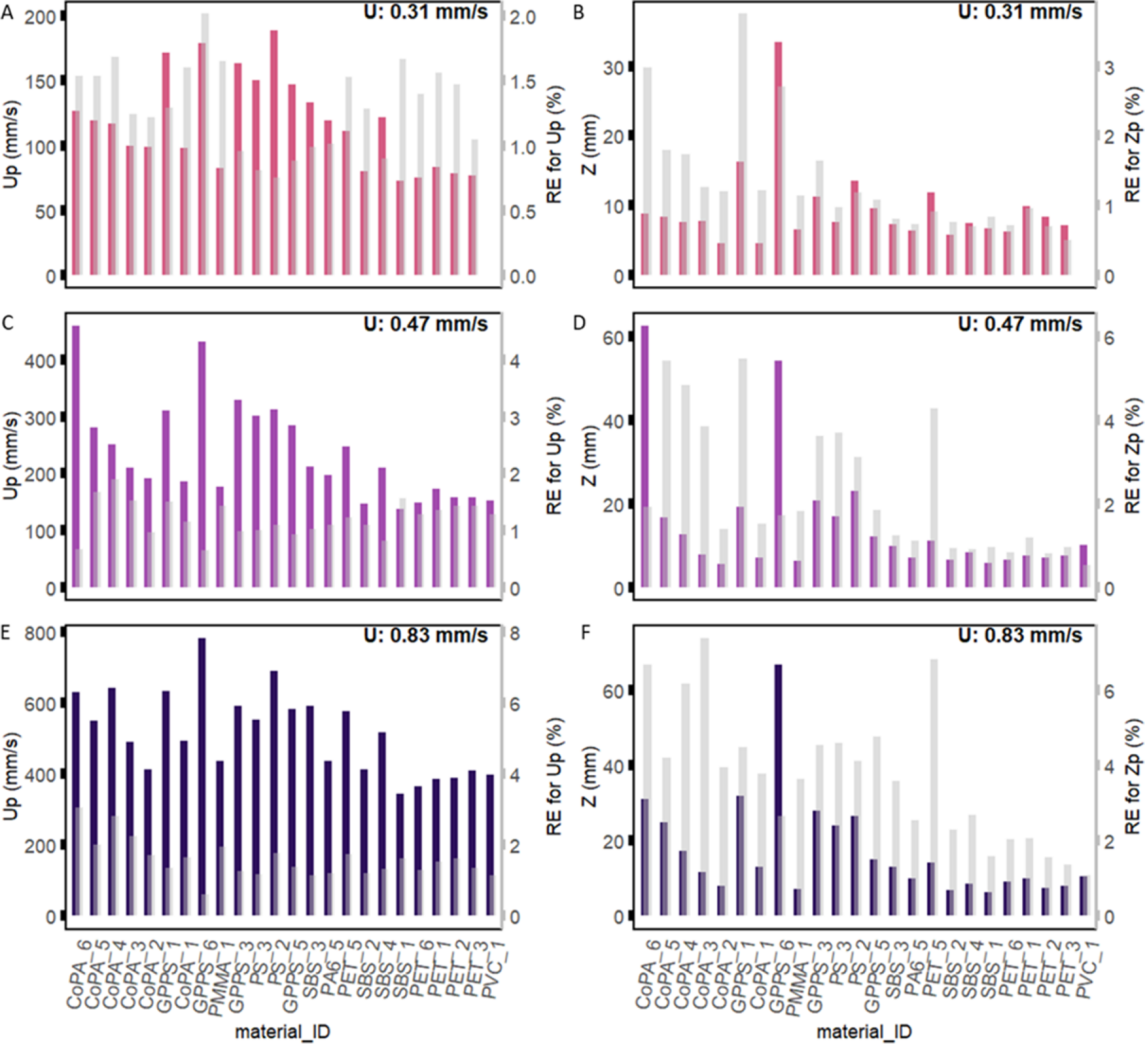
Mean results of the experiments grouped by material type and ordered by D_*_ in ascending order. The mean forward velocity (*U*_P_) (A, C, and E). The mean particle position in the water column (*Z*_P_) (B, D, and F). The gray bars represent the relative error RE (secondary *y*-axis). The three rows show the results of the different water velocities used.

Reproducibility was assessed through the relative error, considering all instantaneous measurements for each flow-particle combination. This comprehensive analysis provided a robust evaluation of the experimental results reliability across various conditions. In Figure 4, the resulting relative errors are presented in an ascending order based on D_*_. The relative error concerning the mean particle position exhibited a range from 0.5 to 7.4%. Overall, the relative error decreased with increasing particle density, suggesting a potential restriction in the randomness of the process for denser particles mostly remaining closer to the bed. Conversely, the relative error increased as the flow increased, which could be attributed to increased scatter introduced by higher turbulence. Lower relative errors were apparent in the mean forward velocity, ranging between 0.61 and 3.05%. An increase in particle density was associated with an increase in the relative error for the mean forward velocity, indicating a higher level of randomness introduced by the additional friction impeding the particle transport and increased particle-bed interactions.

Further, fragmented particles (PET_5) had one of the highest relative errors for the position (up to 6.5%), potentially attributed to their high shape heterogeneities in the present study. Indeed, the three principal dimensions determined for PET_5 had the highest standard deviation, ranging between 40 and 80%. This observation suggests that shape and size are crucial factors in determining microplastics occurrence and transport in rivers.

#### 3.1.2 Trajectory Characteristics: Comparison with Previous Sediment and Microplastic Research

**Figure 5:**
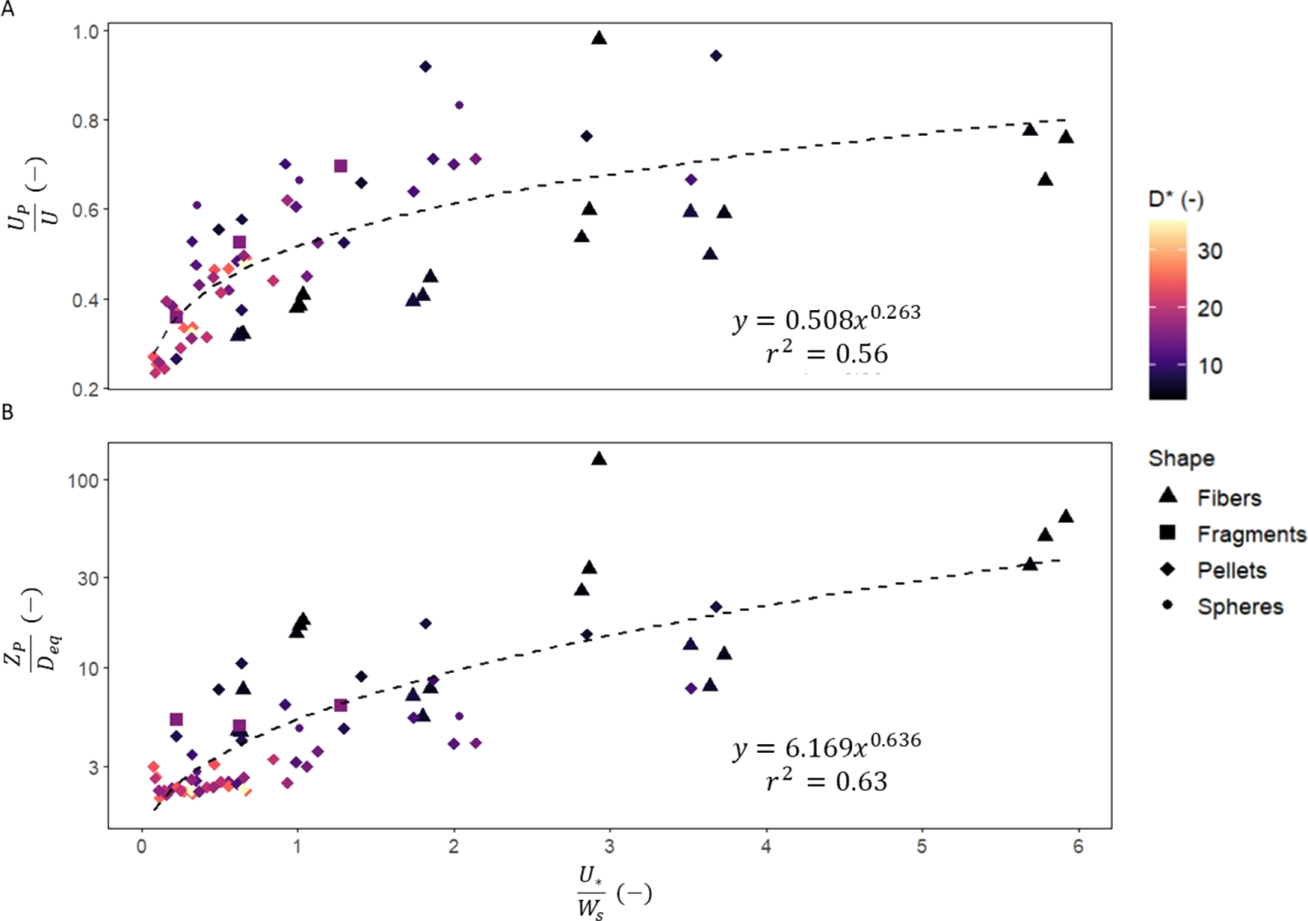
(A) The normalized mean forward velocity of the particles relative to the transport stage and (B) the normalized mean particle position relative to the transport stage. Note that for the normalized mean particle position, an anomaly was observed for the smallest fiber (CoPA_6), necessitating its exclusion from the fit.

In the present study, excluding one anomaly for the smallest fiber (CoPA_6), the particle mean forward velocity (*U*_p_) and position (*Z*_p_), normalized to the flow velocity (*U*) and the equivalent particle diameter (*D*_eq_) respectively, were strongly correlated with the transport stage (u_*_/*W*_s_) with *p* < 0.05 based on a multivariable ANOVA test. Higher transport stages correspond to scenarios with increased relative turbulent forces, influencing the trajectory characteristics. Figure 5 illustrates the strong correlation between the normalized particle trajectory characteristics (*Z*_p_/*D*_eq_, and *U*_p_/*U*) and the transport stage, fitting well to a power function.

These results align with findings for both sediments and microplastics.^14,22−24,41^ The power function proposed by Lofty et al.^14^ for the saltation trajectory of spheres was suitable for the trajectory characteristics in the present study, albeit with lower correlation and different coefficients. The need for modified coefficients is likely because of the particle shape, while the lower correlation could be attributed to multiple factors. The heterogeneous mix of particle transport regimes, combining saltation, suspension and rolling in the trajectory characteristics could explain the discrepancy. For the present study, *Z*_p_ was normalized to *D*_eq_, whereas previous work used the spherical particle diameter for normalization.^14,22^ This approximation of the particle length scale could reduce the correlation for *Z*_p_/*D*_eq_. The forces governing particle transport in the flow are strongly correlated with the surface area.^19^ As the particles deviated from perfect spheres, the fluctuations in the surface area of a rotating particle perpendicular to the flow increased. Consequently, the variations in the force balance increase, leading to lower correlation. Further, the drag coefficient and settling velocity of the particles were determined using equations developed for settling column experiments. The deviation from spherical particles in the present study produces complex relations between the flow condition and the drag coefficient which can significantly affect the transport and settling processes of microplastics.^42^

The results indicated a positive correlation between the particle location (*Z*_P_) relative to the water depth (*h*), measured from the bed, and the normalized mean forward velocity (*U*_p_/*U*). Under the same flow conditions, particles migrating closer to the bed experienced lower velocities. These findings align with Francis & Abbott,^22^ where increased water depth resulted in higher *U*_p_ of saltating particles. The position of the particle relative to the water column can lead to the particle being at different heights within the logarithmic velocity profile which explains the positive correlation between the particle forward velocity and particle position (*r*^2^ = 0.65). In terms of particle position, the water depth was found insignificant in the present study, which deviated from the observations in Francis & Abbott.^22^ Here, the water depth allowed particles to reach maximum height without rebounding from the surface, which could explain the discrepancy.

#### 3.1.3 Impact of Particle Characteristics on the Particle Trajectory

Employing PCA statistical tests indicated that CSF, elongation, sphericity, and aspect ratio are pivotal for *Z*_p_/*D*_eq_, and *U*_p_/*U*, collectively explaining up to 55% of the variance. The second principal component of the PCA is primarily affected by the transport stage and D_*_. A multivariable ANOVA test (*p* < 0.05) showed that elongation significantly affects *Z*_p_/*D*_eq_, while both elongation and aspect ratio significantly impact *U*_p_/*U*. This variation between PCA and the multivariable ANOVA implies a redundancy or overlap between the information conveyed by the different shape factors. This overlap could be explained by the strong correlations among the shape factors as they are all derived from the particle principal dimensions and volume. For both the normalized mean position and forward velocity, D_*_ and the transport stage are significant contributors. The results are illustrated in Figure S4 and Tables S1 and S2 within the Supporting Information.

Despite their statistical significance, the shape factors maintained low regression values individually (r^2^ ranged between 0.005 and 0.35), compared to the transport stage. However, the impact of particle properties can be observed qualitatively in Figure S2, showing a scatter of the trajectory characteristics against the transport stage, where the impact of the particle properties is visualized. *Z*_p_/*D*_eq_, and *U*_p_/*U* decreased as D_*_ decreased, similar to the observations for sediments.^24^ However, relying only on D_*_ proves insufficient in explaining the overall trend. In addition to gravitational forces, two crucial forces, buoyancy and drag, can act on a particle moving in water. These forces are intricately linked to the particle’s surface area, which, in turn, is influenced by its geometry.^19^ Notably, fibers exhibit a higher susceptibility to suspension in the water column compared to fragments and spherical particles, due to their increased buoyancy and reduced settling velocity.^43^ In our study, fibers reached the highest position in the water column (Figure 5B), aligning with an inverse correlation between *Z*_p_/*D*_eq_, and sphericity and elongation. The lift force, influenced by the particle’s surface area, tends to push the particle higher in the water column as it deviates from a perfect sphere.

In terms of the velocity, fibers had lower *U*_p_/*U* compared to spheres (Figure 5A). This can be attributed to two key factors. Spherical particles are more prone to entrainment from the bed compared to fibers, where the particle entrainment potential is expected to increase as sphericity approaches 1.^19,44^ Spherical particles experience less friction once in contact with the bed, leading to reduced kinetic energy loss during rolling or saltating. Moreover, the migration of nonspherical particles is highly influenced by their orientation and rotation regime.^45^ Fibers tended to exhibit an oscillating velocity, moving up and down, which could explain the large relative error for the mean forward velocity of fibers (Figure 4B). Thereby, fibers needed more time to travel through the observation window, which reduced their average mean forward velocity.

### 3.2 Modes of Transport: The Phase Diagram

In this study, all particles exhibited mobility, except for the pellets with a lenticular shape (PVC_1), which remained stationary under the lowest flow condition. Unsurprisingly, D_*_ emerged as a crucial factor determining the three distinct transport modes, as shown by both the multivariable ANOVA and PCA tests (see Tables S1 and S2 and Figures S3 and S4). Higher D_*_ values correlated with bedload transport, whereas decreasing D_*_ values were associated with an increased duration of particle suspension. Further, the particle shape had a pronounced effect on the particle transport mode. Spherical particles exhibited susceptibility for rolling and saltation, while fibers demonstrated a proclivity for suspension. The PCA test showed that the particle shape factors CSF, sphericity, elongation, and aspect ratio were the primary contributors. The multivariable ANOVA test further confirmed the significance of the particle shape for the three modes of transport. Shape parameters, specifically elongation, had a statistically significant influence on particle suspension behavior. For bedload transport modes, encompassing saltation and rolling/sliding regimes, the particle shape factors CSF and elongation were found to be statistically significant contributors. The findings highlight the interplay between particle geometry, surface area, and governing forces in particle transport, aligning with previous sediment^46^ and microplastic research.^19^ For the saltation mode, the effect of the particle shape can play an additional role in the characteristics of the collision with the bed and the associated energy losses induced by the impact and the rebound dynamics.^47−49^ Spherical particles have a constant projected area in contact with the bed at any collision point, which reduces heterogeneities in the collision and the associated energy losses. The contact area for other particles tends to vary over time, increasing the randomness of the collisions and reducing the predictability of particle transport mode.

**Figure 6:**
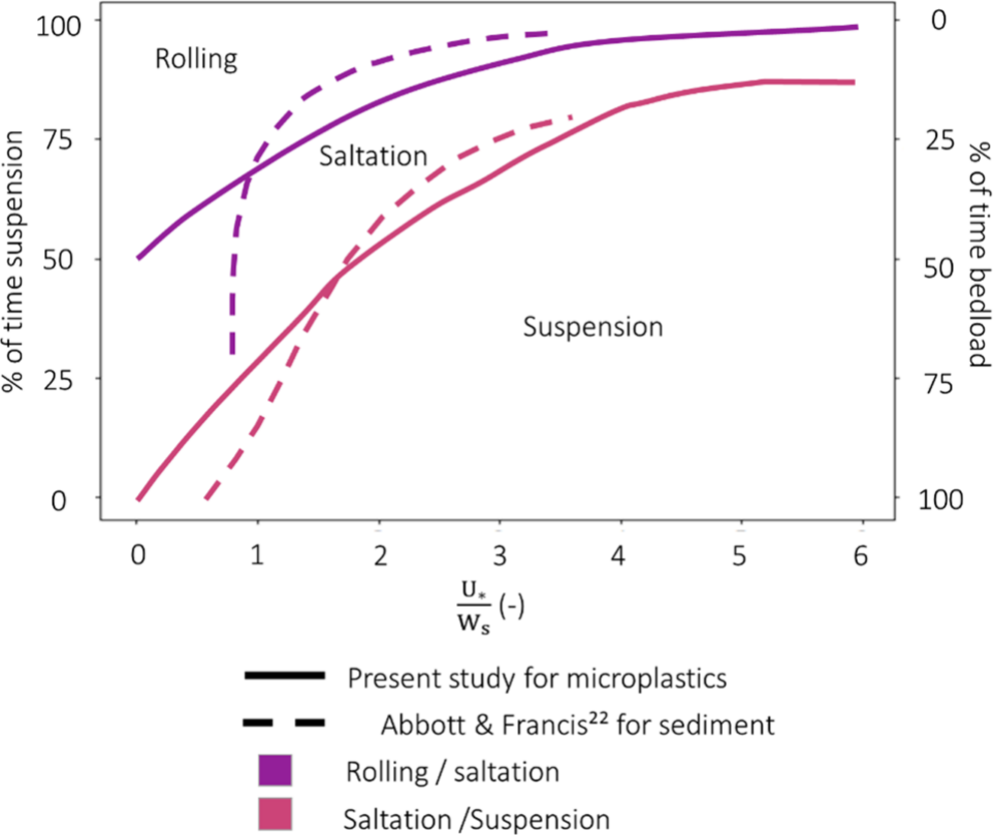
Resulting phase diagram for microplastics compared to the one for sediment.

The modes of transport exhibited by the particles appeared to be correlated with the transport stage (see Figure S5 in Supporting Information). These transport modes are within the same order of magnitude as those for perfect spheres in Lofty et al.,^14^ despite variations in bed material grain size and shear velocities. Due to the irregularly spaced nature of the collected data, the fit was determined based on a locally weighted regression algorithm (LOESS).^50,51^ The analysis was performed following the method described in de Ruijsscher et al.^52^ It should be noted, however, that the multivariable ANOVA test suggested that the transport stage is not a significant factor governing particle saltation, possibly due to the previously discussed randomness of collisions for nonuniform particles (Table S2). The result of the present study allows for the generation of a phase diagram to predict the modes of transport experienced by a microplastic particle as a function of the transport stage (Figure 6).

### 3.3 Assessing the Phase Diagram against Previous Sediment and Microplastic Research

In the present study, microplastics experienced initiation of motion at lower transport stage values compared to natural sediment, leading to discrepancies in the resulting phase diagram (Figure 6). This difference may be attributed to their lower critical shear stress, as indicated in the findings of Waldschläger and Schüttrumpf^19^ and Goral et al.^53^ Moreover, the curve fitting for microplastics displays a less steep slope, particularly for transport stage values below 2, in contrast to sediment dynamics. Overall, the resulting phase diagram suggests that while the concept of transport stage can be adapted from sediment studies, adjustments are necessary to accommodate for the unique characteristics of microplastics.

Similar microplastic phase diagrams in the literature are limited to the one developed by Huang et al.,^18^ where the concentration depth profile of four microplastics was numerically modeled. However, Huang et al.^18^ focused on density driven turbulence mixing and did not account for the dynamics of the bottom boundary, which is a primary focus of the present study. Additionally, the scope of their results was limited to three particle diameters, which make a direct comparison with the phase diagram presented here not possible.

While the bed roughness had a significant impact on the saltation and rolling/sliding of spherical particles in previous studies,^14^ this impact was negligible in our study. This discrepancy could be due to particle shape affecting the hiding-exposure effect. For instance, fibers were more prone to being trapped in the bed material than spherical particles.^15^ Moreover, previous sediment studies suggested that bed roughness becomes inconsequential for particle suspension when the ratio of particle diameter (d_p_) to bed material diameter (d_b_) exceeds 0.5.^23^ This observation elucidates the insignificance of bed roughness for particle modes of transport in our study, given that all examined particles had a d_p_/d_b_ ratio greater than 0.5.

### 3.4 Study Limitations and Outlook

The presented research offers valuable insights into microplastic transport dynamics, yet further research is needed in the future. To improve the robustness of our findings, the range of particle shapes, sizes, and densities could be further expanded. The presented results and the proposed phase diagram do not cover buoyant microplastics. Their transport dynamics differed significantly from nonbuoyant microplastics at the particle scale, necessitating dedicated experimental setups. Further, the presented results are limited to the size ranges included in the study (i.e., 0.5–5 mm). Smaller particles in particular might exhibit different transport characteristics, such as those observed in suspended matter or cohesive sediment. Therefore, the presented results could be improved by examining a wider range of particles sizes. Due to the limited shapes in the adopted material matrix, fibers dominated at transport stage values ≥3, potentially skewing results and interpretations. To improve robustness, examining a wider range of shapes, including fragments, pellets and fibers, particularly for transport stage ≥3, is recommended. Although the effect of particle shape was statistically significant, correlations with microplastic transport were low (*r*^2^ < 0.35). Expanding the sample size to include a wider range of shapes may improve these correlations, which could lead to a more robust mathematical representation of the effect of the particle shape on microplastic transport in transport models. Moreover, the effects of riverbed material, collision dynamics, biofouling, coagulation, water chemistry, and texture were excluded in this study. Future research should further investigate such processes and factors governing plastic transport to better reflect real-world conditions.

The determination of the transport stage demanded the particle settling velocity as an input, which was calculated following Waldschläger & Schüttrumpf.^15^ The particle shape dictates the settling velocity (*W*_S_) and the drag and friction coefficients governing particle motion. Given the abundance of fragments in water and sediments^54^ and the uncertainty in incorporating the effect of shape to calculate these coefficients,^20^ further research is needed to accurately account for irregular shapes.

The experimental setup could be further improved. The setup accuracy could be enhanced by incorporating the effect of refraction in the camera calibration algorithm. The automated method for analyzing the particle trajectories was more effective for spherical particles than irregular shapes. The need for visual inspection highlights its limitations in fully capturing trajectory complexities due to particle rotation. Future work should target an advanced trajectory analysis approach to account for the change in the particle dimension facing the camera to enhance accuracy and reliability, particularly for nonuniform particle shapes.

Lastly, despite their importance, particle-scale experimental investigations like this study should be coupled with descriptive models that account for the full range of particle properties. This approach would enhance understanding of microplastics’ behavior in the environment. Further, integrating laboratory experiments, similar to our work, into computational fluid dynamics (CFD) models has shown promising results to improve hydrodynamic microplastic transport models.^18,55,56^ However, so far, near-bed interactions have not been included in such models. Aided by our generated data set, future modeling effort could further expand present transport models to account for the bed-particle interactions. An additional attractive option is to explore machine learning tools capabilities.^57^ For example, a machine learning model was used for modeling microplastics settling velocity.^58^ Further exploration of such models for microplastic transport studies could lead to significant advancements.

### 3.5 Environmental Implications of the Results

The present study examined microplastic transport at the particle scale. The effects of key particle properties (e.g., shape and D_*_) influencing the particle transport in rivers were examined. The proposed transport stage offers a simplified approach to include turbulence into hydrodynamic models, allowing for determining microplastic transport rates and potential hotspots along the water column. In practice, the results indicate that the transport stage is a suitable predictor of microplastic transport modes, and that the modes of microplastics are comparable to those of sediment. However, at lower transport stages (i.e., lower shear velocity and/or higher settling velocities) microplastics exhibit saltation and suspension whereas sediments are transported as bedload, resulting in a higher proportion of particles present in the water column compared to sediment under these conditions. The incorporation of the particle shape effect improves the interpretation and analysis of sampling data. Fibers were dominant in the upper part of the water column while spheres were predominantly transported near the bed. This could be applied to enhance the estimations of microplastic from a single-point measurement along the water column. The derived mean position can guide appropriate measures, such as barriers or cleanup operations, targeting the most probable depths for microplastic presence. It also has important ecological applications. Knowledge about the dominant microplastics at various depths relative to the organisms inhabiting such areas facilitates evaluating pollution risks. In conclusion, we propose that the transport stage can be used to predict the behavior of nonbuoyant microplastics in rivers. Due to its potential to improve transport prediction models, exposure assessments and interventions strategies, it facilitates targeted cleaning and microplastic pollution collection in riverine ecosystems.

## Data Availability

The data set is available in the Supporting Information. The image analysis code can be found in the following Open Science Framework link https://osf.io/6g8fj/?view_only=6fe6d462447f40108a35512a01ef4d72

## Supporting Information Available

Layout of material properties; visual inspection of the effect of the particle shape, statistical analysis of the effect of the particle shape; and the derivation of the phase diagram (PDF)
Raw data for particle analysis (XLSX)
