# Correction to “Mapping Microplastic Movement: A Phase Diagram to Predict Nonbuoyant Microplastic Modes of Transport at the Particle Scale”

**DOI:** 10.1021/acs.est.6c05935

**Published:** 2026-05-23

**Authors:** Hadeel Al-Zawaidah, Merel Kooi, Ton Hoitink, Bart Vermeulen, Kryss Waldschläger

In Table 2, the Froude number equation should read $\frac{u}{\sqrt{gh}}$ instead of $\frac{u}{gh}$ , and the kinematic viscosity of water should be denoted as ν instead of u. Additionally, the reported Reynolds number (Re) values for flow regimes F1, F2, and F3 are missing one zero. The correct values are 144420 for F1 (instead of 14442); 108100 for F2 (instead of 10810), and 75330 for F3 (instead of 7533).

In the raw data set for particle analysis in the Supporting Information, the water depths for F1, F2, and F3 were mismatched. The correct values, consistent with Table 2 in the main article, are 17.4 cm for F1; 23.0 cm for F2; and 24.3 cm for F3.

These corrections do not affect the results, interpretation, or conclusions of the paper.

## Supplementary Material



## Notes

Raw data for particle analysis (XLSX)

