# Supplementary material for: Mapping Microplastic Movement: A Phase Diagram to Predict Nonbuoyant Microplastic Modes of Transport at the Particle Scale
Source: Environ Sci Technol. 2024 Sep 28;58(40):17979–89. doi: 10.1021/acs.est.4c08128 (PMC11465633; doi:10.1021/acs.est.4c08128)
Supplement: Supplementary file 1 — es4c08128_si_001.pdf [file es4c08128_si_001.pdf]

Supporting information to:
Mapping microplastic movement: A phase diagram to predict non-buoyant microplastics modes of transport at the particle scale

Hadeel Al-Zawaidah[1*], Merel Kooi[2], Ton Hoitink[1], Bart Vermeulen[1] & Kryss Waldschläger[1]

[1]: Wageningen University and Research, Hydrology and Environmental Hydraulics Group, 6700 AA Wageningen, The Netherlands
[2]: Wageningen University and Research, Aquatic Ecology and Water Quality Management Group, 6700 AA Wageningen, The Netherlands

[*]Email: hadeel.alzawaidah@wur.nl

Summary of Supporting Information Contents:

- Number of pages: 5
- Number of figures: 5
- Number of tables: 2
- Number of Excel files: 1 (Raw data for particle analysis: Excel file)

Table of Contents

S1) Material properties S2

S2) Visual inspection of the effect of the particle shape S3

S3) Statistical analysis of the effect of the particle shape S4

S4: Derivation of the phase diagram S4

S1) Material properties

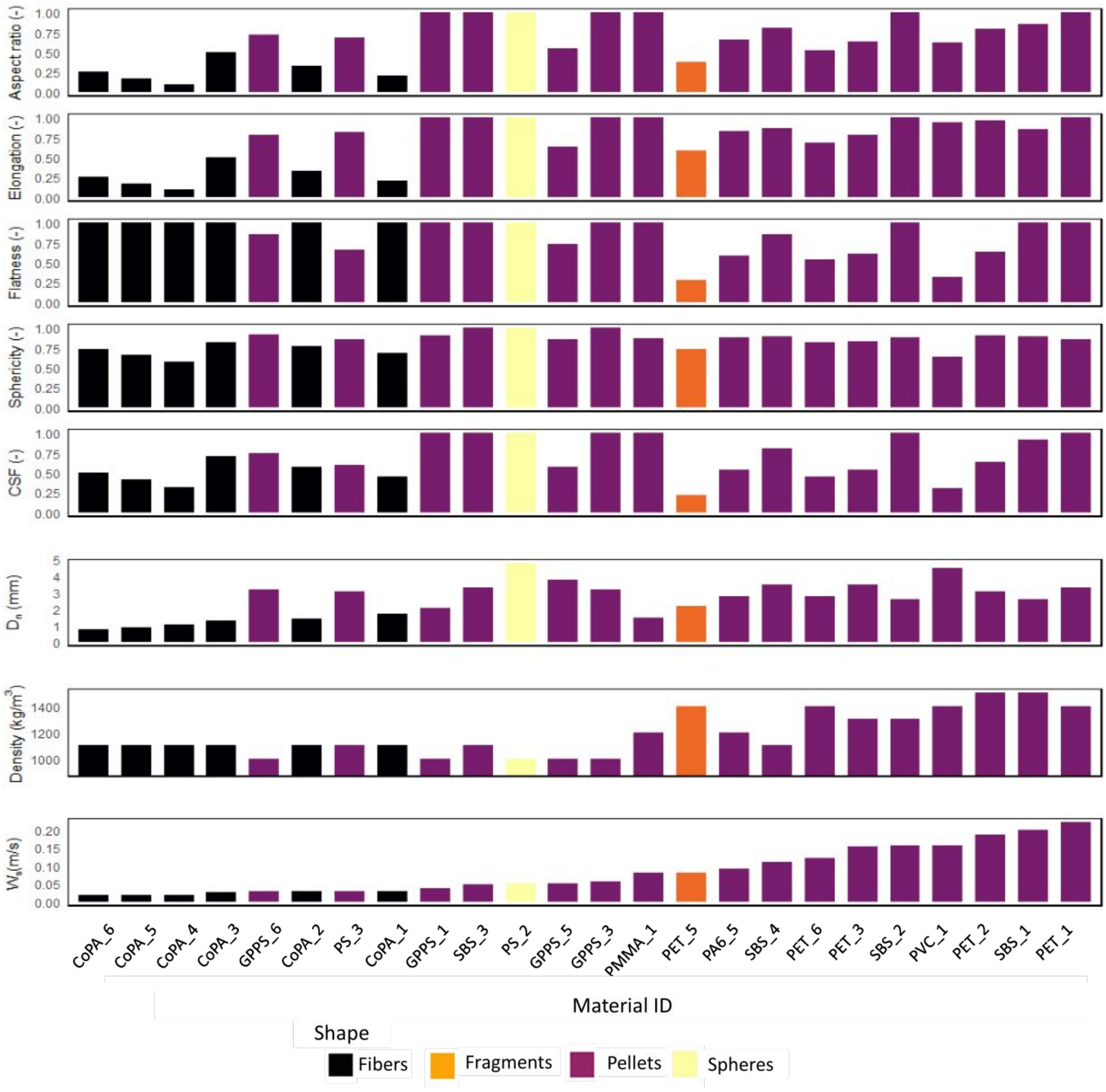

Figure S1: Layout of the particle properties. The abbreviations in the particle names refer to the main polymer formulating the particles. CoPA is Nylon , GPPS is General Purpose Polystyrene, PS is Polystyrene, PMMA is Poly(methyl methacrylate), PET is Polyethylene terephthalate, PVC is polyvinyl chloride, SBS is Poly(styrene-butadiene-styrene)

S2) Visual inspection of the effect of the particle shape

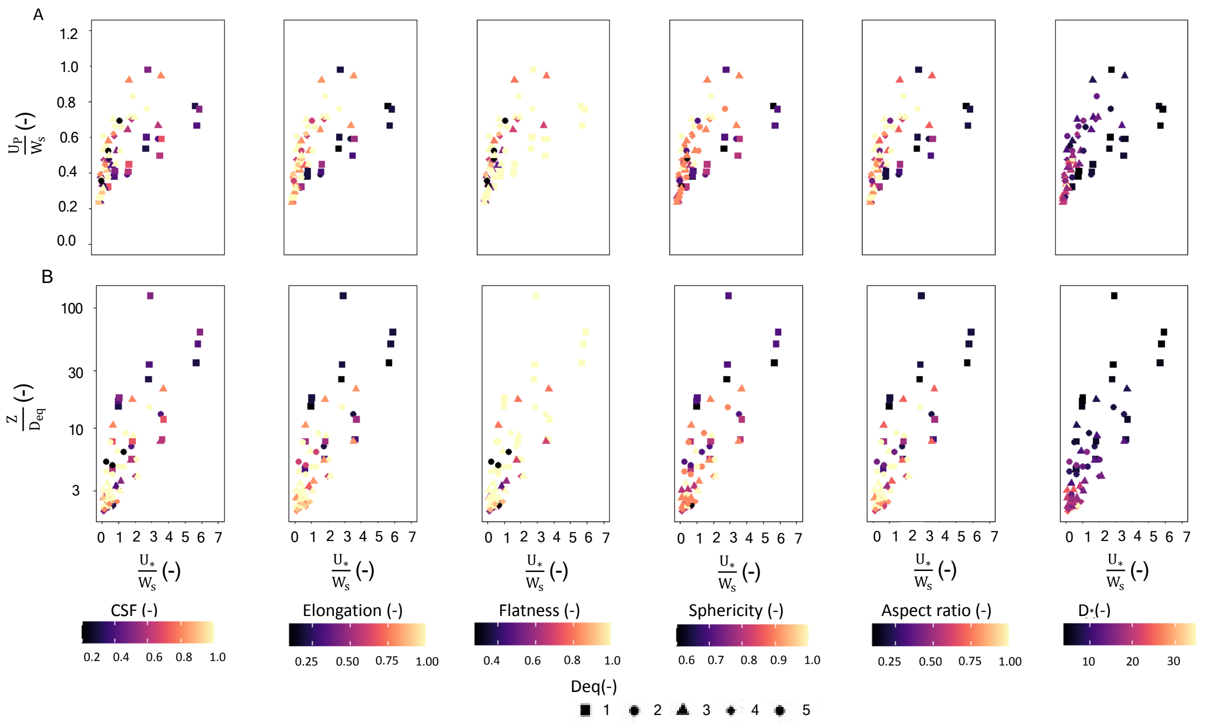

Figure S2: Layout of the particle trajectory characteristics faceted by microplastics characteristics.

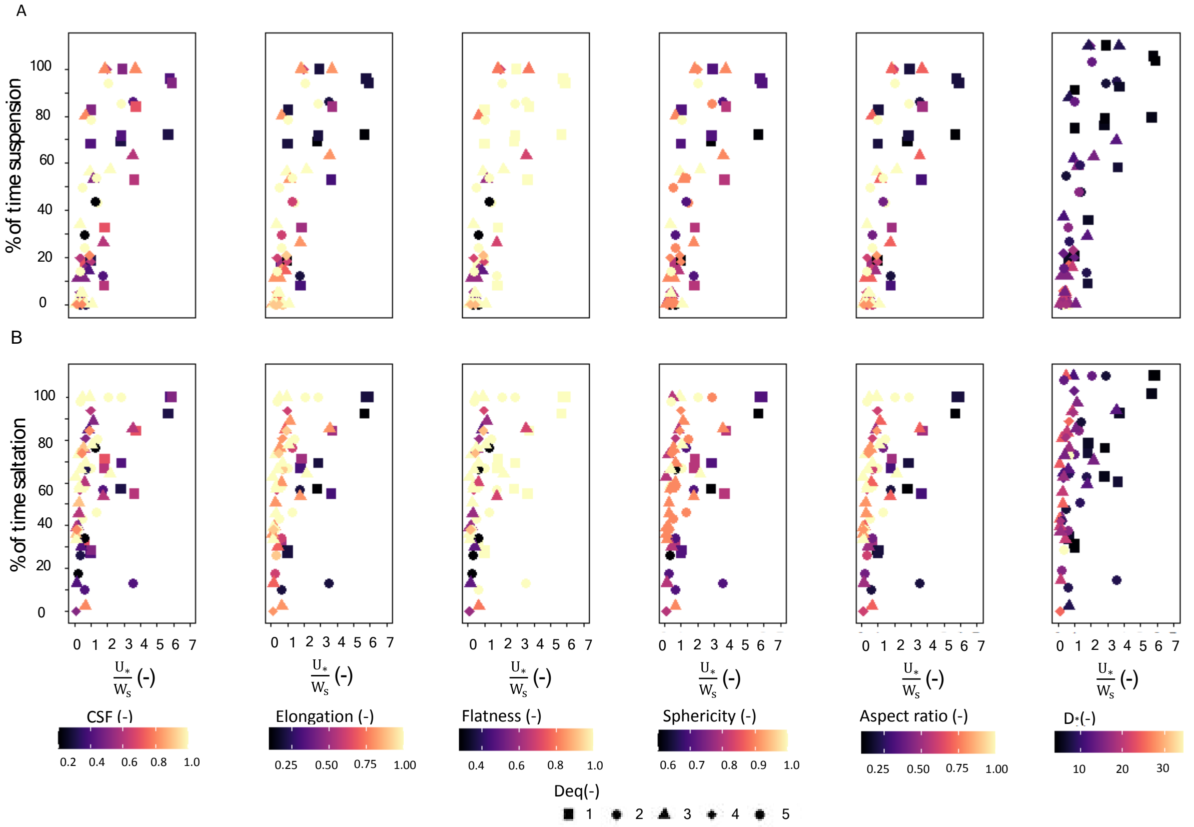

Figure S3: Layout of the particles modes of transport faceted by microplastics characteristics.

S3) Statistical analysis of the effect of the particle shape

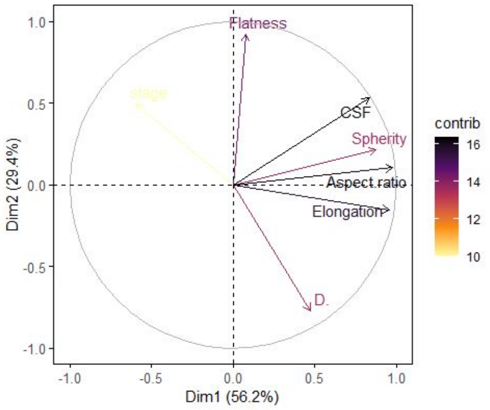

Figure S4: Graphical representation for the PCA results in the two primary principal components.

Table S1 : PCA loading results for the predictor variables.

Parameter	PC1	PC2	PC3
CSF	0.42083961	0.37114203	-0.08036899
Sphericity	0.44300254	0.15163019	0.23729534
Elongation	0.48419153	-0.10847351	0.24345468
Flatness	0.03893025	0.64223866	-0.39129138
Aspect ratio	0.49432135	0.07275268	0.13119666
D_*	0.23813821	-0.53861589	0.05266317
$\frac{u_*}{W_S}$	-0.29936994	0.34586826	0.83956337

Table S2 : Summary of the ANOVA test results.

Parameter	P value				
	$\frac{Z}{D_{eq}}$	$\frac{U_P}{U}$	% of time in suspension	% of time in saltation	% of time in rolling/sliding
CSF	0.120909	0.607662	0.3883	0.00687	0.035184
Sphericity	0.073360	0.092176	0.4815	0.54678	0.857247
Elongation	0.001217	0.000346	9.55e-09	0.00185	0.000497
Flatness	0.411414	0.129317	0.0777	0.62975	0.132576
Aspect ratio	0.798507	0.048424	0.1643	0.82737	0.209002
D_*	0.009648	4.23e-07	1.10e-07	0.00193	0.002448
$\frac{u_*}{W_S}$	0.000849	8.65e-11	2.05e-08	0.16333	3.13e-07

S4: Derivation of the phase diagram

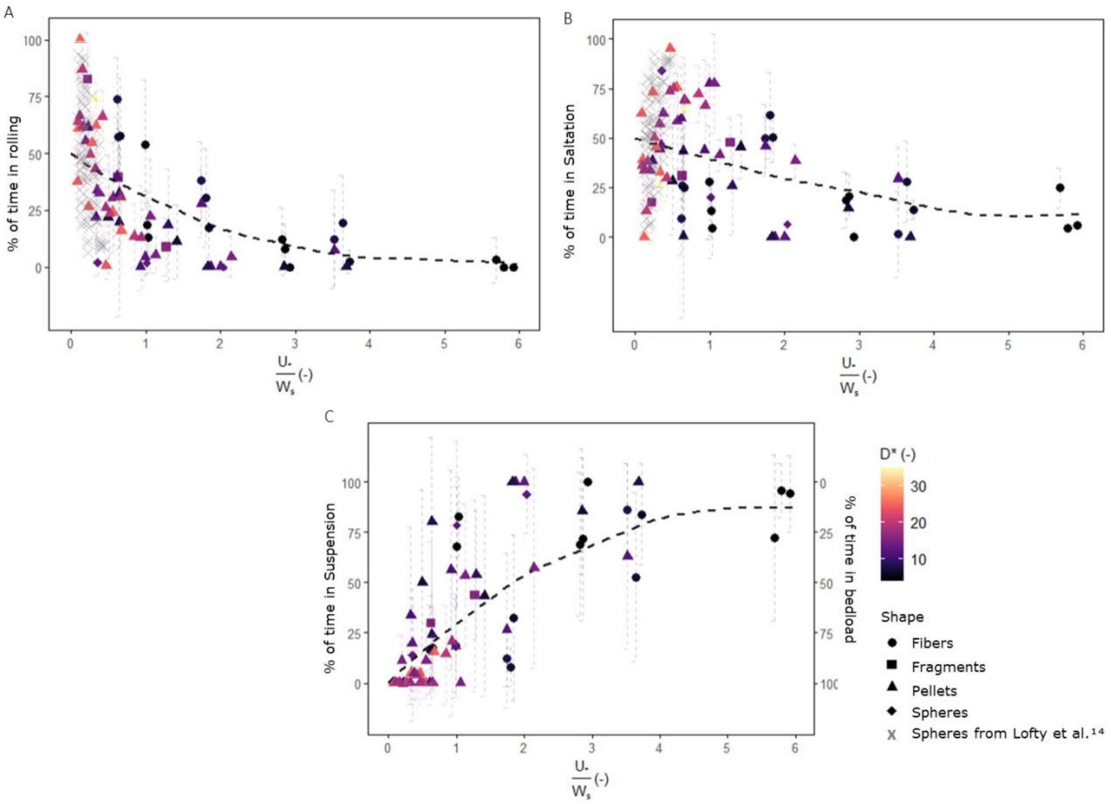

Figure S5: The percentage of time a particle exhibited rolling against the stage, A, the percentage of time a particle exhibited saltation against the stage, B, and the percentage of time a particle exhibited suspension against the stage, C.
